# Internal Dialogue as a Mediator of the Relationship Between Prayer and Well-Being

**DOI:** 10.1007/s10943-019-00943-2

**Published:** 2019-11-09

**Authors:** Małgorzata M. Puchalska-Wasyl, Beata Zarzycka

**Affiliations:** Institute of Psychology, The John Paul II Catholic University of Lublin, Al. Racławickie 14, 20-950 Lublin, Poland

**Keywords:** Upward prayer, Inward prayer, Outward prayer, Internal dialogues, Well-being

## Abstract

When people are praying, they are not only communicating with God (upward prayer), but also they are exploring their relationships with themselves (inward prayer) and with other people (outward prayer). Internal dialogical activity includes areas which correspond to upward, inward, and outward prayer. Therefore, the aim of this article is to examine whether and how internal dialogues can be mediators in the relationship between these three types of prayer and well-being. Data from 193 respondents (143 females) were analyzed in the study. We used: the Prayer Thoughts Scale, the Internal Dialogical Activity Scale, and the Psychological Well-Being Scale. The results showed that internal dialogue served as a mediator of the relationship between upward, inward, and outward prayer and well-being.

**Electronic supplementary material:**

The online version of this article (10.1007/s10943-019-00943-2) contains supplementary material, which is available to authorized users.

## Introduction

James (1902) asserted that prayer is the very soul and essence of religion. Prayer is communication through which one relates to and even identifies with God. According to James (1902, p. 464), prayer is “every kind of inward communion or conversation with the power recognized as divine (…).” The communicative aspect of prayer seems to be its most evident feature (Baesler 2012; Spilka and Ladd 2014). Baesler (1997), reviewing themes of 50 definitions of Christian prayer, indicated that some form of “communication,” such as talking, listening, sharing, and dialoguing, is essential to defining prayer. Moreover, most major world religions include some type of communication as a defining characteristic of prayer (Baesler 2012). Academic disciplines that investigate prayer as a religious phenomenon build on the assumption that “communication” is integral to understanding prayer (Baesler 2012).

In psychology, Ladd and Spilka (2002, 2006) proposed a multidimensional model of prayer based on the work of Foster (1992). According to Foster, the purpose of prayer is to connect the person praying to some type of specific reality where prayer is directed in three directions. First, prayer can be directed inward and focus on self-examination. Second, prayer can be directed outward and focus on relationships with others. Finally, prayer can be directed upward and focus on the relationship between the believer and the divine (Lazar 2017). Ladd and Spilka (2002, 2006) developed a program of psychological research examining the cognitive aspects of prayer as connection with the divine (upward), the self (inward), and with others (outward). Upward prayers explicitly seek to connect the person praying with the divine. The desire to find quietude or stillness in the presence of God is typical of the prayer of rest, which is the classic example of upward prayer. Inward prayers serve to connect a person with his or her own spiritual condition in light of chosen theological positions. One form of inward prayer is an examination of conscience, an intellectual cataloguing of the ways in which one has lived up to or fallen short of theological tenets. Outward prayers are characterized by their emphasis on connecting with different facets of physical relational life. The essence of outward prayer is the intentional desire to join in the suffering of another person (Ladd et al. 2007).

In recent decades, there has been a tremendous increase in scientific interest regarding the links between prayer and well-being, but the results of this research are inconsistent. Most findings show positive relationships between prayer and subjective well-being (e.g., Carroll 1993; Francis and Evans 1996; Richards 1991). There are also studies which have failed to find a relationship between prayer and well-being (e.g., Ellison et al. 2001; Markides 1983). Finally, there are studies which found that some types of prayer had negative links with well-being (e.g., Poloma and Pendleton 1991). Such inconsistency suggests that the relationship studied might depend on other variables that act as moderators or mediators. A variable that can be mediator in the relationship between prayer and well-being is internal dialogical activity. It includes three areas, namely (1) internal dialogues (IDs) with figures who are not part of our social environment (e.g., with a personal God); (2) juxtaposing of the viewpoints relevant for personal and/or social identity; and (3) IDs with people we personally know (e.g., with a friend) (Puchalska-Wasyl et al. 2008). These areas seem to correspond to the upward, inward, and outward prayer, respectively. Such correspondence lets us assume that IDs can be involved in prayer.

Although prayer can quite often be accompanied by ID, we assume that they are distinct phenomena—prayer cannot be reduced to any type of ID. Indeed, on a behavioral level we can observe some similarities between prayer and ID (e.g., talking to someone whom we cannot see, saying what’s on our mind, etc.). However, at the phenomenological level there is a profound difference between them. A partner in an ID is “only” an important viewpoint. For example, when I engage in an ID with my friend, he or she has no access to this imagined activity. Prayer is something different. The deity in prayer is perceived as a metaphysical “other” with qualitatively distinct features like omniscience (Ladd et al. 2012), thanks to which God knows every human behavior and thought, and thus, He knows the content of prayer. On the other hand, God by His qualitatively distinct features is so different from human being, that a person can feel God is distant. Treating God as a partner or listener of ID makes Him closer in psychological sense. Therefore, we are of the opinion that personal prayer can be a starting point for ID in which God becomes a partner or listener. In this sense, on an observational level prayer can take a form of intrapersonal communication.

In this context, the aim of this article is to examine whether and how IDs can be mediators in the relationship between upward, inward, and outward prayer and well-being. Before our hypotheses are put forward, we will present the results of research on three basic variables: prayer, well-being, and ID.

### Prayer and Well-Being

There are many studies that show positive relationships between prayer and different measures of subjective well-being. For example, Richards (1991), in a sample of 345 people, found a positive correlation between the intensity of the prayer experience and self-reported purpose in life. Similarly, in a study of 100 members of Alcoholics Anonymous, Carroll (1993) noticed a positive correlation between purpose in life and a variety of spiritual practices, including prayer. Francis and Evans (1996) also explored this relationship, analyzing two samples of 12- to 15-year-olds. The first sample comprised 914 males and 726 females who never attend church. The second sample comprised 232 males and 437 females who attend church most weeks. The data showed a positive relationship between frequency of personal prayer and perceived purpose in life for both groups. Additionally, Francis (1992) explored the relationship between prayer and attitude toward school among a sample of 3762 11-year-old pupils. After controlling for individual differences in church attendance, Francis found that pupils who prayed reported a more positive attitude toward school.

Another study was conducted on a sample of 474 college students in the UK. Maltby et al. (1999) analyzed the association between frequency of prayer and three measures of well-being (depression, trait anxiety, and self-esteem). In the group of women, as well as in the group of men, frequency of prayer predicted lower depression, lower anxiety, and greater self-esteem. Carlson et al. (1988) compared levels of anxiety and anger between three experimental groups, each containing 12 college students. The first group followed a program of prayer and biblical meditation, the second group followed a program of progressive relaxation exercises, and the third group was the control. After a two-week period, members of the first group reported less anger and anxiety than members of the other two groups. Krause (2004) studied self-report data from a sample of 1258 white and African-American adults at least 66 years of age. It was found that self-esteem was highest when respondents believed that only God knows when and how to best answer prayer. Among those participants who expected prayers to be answered immediately and believed that they get what they ask for, self-esteem was lower.

Poloma and Pendleton (1991) found that different prayer types had different links with general well-being. The authors identified four types of prayer using a factor analysis of responses to an interview survey with 560 participants. Meditative prayer reflected intimacy and personal relationship with the divine and was described by words such as adoring, reflecting, and communicating. Ritual prayer was understood as a recitation of prepared prayers available through readings or memory. Petitionary prayer was defined as requesting that specific material needs are met for self and friends. Finally, a conversational style of prayer that incorporates petitionary elements but is less concrete and specific was called colloquial prayer. Poloma and Pendleton observed that meditative prayer was significantly related to existential well-being and religious satisfaction and that colloquial prayer predicted happiness. However, ritual prayer predicted greater depression, loneliness, and tension.

Other research has failed to find a relationship between prayer and well-being. For example, Ellison et al. (2001), in their study on 1139 American adults, initially observed a weak negative relationship between frequency of prayer and well-being, but the association was reduced to being statistically nonsignificant when social stressors were controlled. Markides (1983) and Markides et al. (1987) failed to find a relationship between prayer and life satisfaction in their longitudinal study. Similarly, Koenig et al. (1993) did not confirm a significant relationship between prayer or bible study and anxiety symptoms in a sample of 1299 adults aged 60 years and above.

Inconsistent results in research on the relationship between prayer and well-being can be due to the fact that not only are different types of prayer taken into account and different groups studied, but also that well-being is understood in different ways and different measures of it are used. The individual pursuit of well-being has been studied through two perspectives: hedonistic and eudemonistic. According to the hedonistic view (propagated by Epicurean philosophy), pleasure is the main source of happiness. In accordance with the eudemonistic approach (typical for Aristotelian philosophy), happiness is the result of engaging in valuable goals (cf. Oleś and Jankowski 2018).

Hedonistic and eudemonistic approaches have been combined in the Authentic Happiness Theory by Seligman (2002). He has posited three distinct pathways to well-being: pleasure, engagement, and meaning. The study on a group of 13,565 participants conducted by Schueller and Seligman (2010) showed that all three pathways correlated with higher levels of subjective well-being. However, pursuing engagement and meaning was more strongly related to subjective well-being than pursuing pleasure. Objective indicators of well-being, such as measures of occupational and educational attainment, displayed a slightly different pattern—engagement and meaning were positively related, whereas pleasure was negatively related. Although these results are merely correlational, the researchers are of the opinion that engaging and meaningful activities may have stronger influences on well-being than pursuing pleasure. Thus, the models of eudemonistic psychological well-being seem to be especially worth empirical investigation in the context of prayer. Therefore, in the current study we employed Ryff’s (1989) model of eudemonistic well-being.

### The Nature of Internal Dialogues

Inconsistent results in research on the relationship between prayer and well-being can also mean that the relationship in question is modified by moderators and mediators. As we have mentioned previously, ID can act as a mediator in the relationship between prayer and well-being. What is ID? We assume that a person engages in ID when he/she adopts (at least) two different viewpoints in turn, and the utterances formulated (internally/silently/in one’s mind or externally/aloud) from these viewpoints respond to one another (Hermans 2003; Puchalska-Wasyl 2006, 2016a, b). In this sense, ID is one kind of intrapersonal communication. The concept of ID is strongly rooted in Dialogical Self Theory (DST; Hermans 2003; Hermans and Gieser 2012), according to which dialogical relationships exist not only between the self and others but also within the self. Traditionally, self refers to processes taking place within the individual mind (“internal”), whereas dialogue is understood as a communicative process between two or more people (“external”). The concept of dialogical self transcends the internal–external dichotomy by bringing the external to the internal, and vice versa. This allows for studying not only society as inhabited by selves of individual people but also the self as a society of mind. “Like people have positions in organized society, they are populated by I-positions in the organization of their own selves” (Hermans et al. 2018, p. 7). As a result, dialogical self is defined as a dynamic multiplicity of relatively autonomous I-positions that represent different points of view available to a person. Each I-position, shaped by a particular social context, is endowed with a voice (the voice of a culture, community, significant other, or one’s own voice) and intertwined with other I-positions, resembling people in social relationships (Hermans 2003). Consequently, not only external/interpersonal but also internal/intrapersonal dialogues are possible. The viewpoints adopted in ID can represent both personal perspectives (e.g., “I-as a believer”) and someone else’s perspectives (e.g., God’s viewpoint).

ID is a heterogeneous phenomenon; therefore, it fulfils different functions. One distinction has been made between integrative and confrontational IDs with respect to their mode and outcome. Integrative IDs aim to integrate all the viewpoints involved; consequently, they can result in creative solutions. Confrontational IDs, by contrast, stress differences between standpoints and aim to enhance one of them and ignore or depreciate the others (cf. Borawski 2011; Młynarczyk 2011; Nir 2012; Puchalska-Wasyl 2016b). Comparison between these two types of IDs showed that integrative IDs perform key functions of support, bond, insight, and self-guiding to a greater degree than confrontational IDs (Puchalska-Wasyl 2016a).

Additionally, IDs can be classified with respect to the functions they serve (cf. Puchalska-Wasyl 2007). In Oleś’s (2009) proposition, which will be discussed in more detail later (see “Measures” section), identity dialogues, supportive dialogues, ruminative and confronting dialogues, among others, are distinguished. Identity dialogues aim at better self-knowledge and at answering identity questions. Supportive dialogues confirm the possessed beliefs and provide a sense of being understood by the imagined interlocutor. Ruminative dialogues are focused on unpleasant topics that invoke feelings of weariness, frustration, and internal breakdown. Finally, when a dialogue involves two clearly separated parts of the self that are in conflict, we call this a confronting dialogue.

### The Current Study

In the context of mentioned studies, prayer seems to be associated with well-being (e.g., Carroll 1993; Francis and Evans 1996; Poloma and Pendleton 1991; Richards 1991). Studies on the function of IDs also suggest that ID can be linked with well-being. In light of definitions by Oleś (2009), ruminative and confronting IDs appear to be non-adaptive. We also know that both these types of IDs correlate positively with anxiety, ruminative IDs correlate negatively with secure attachment style, and confronting IDs correlate negatively with self-esteem (Oleś et al. 2010), and thus, we can infer that these types of IDs are negatively related to well-being. At the same time, supportive and identity IDs correlate positively with secure attachment (Oleś et al. 2010), which allows us to think that they can be positively associated with well-being. Given that and our additional assumption that personal prayer can be a starting point for ID in which God becomes a partner or listener, we posed the following hypotheses:

#### H1

Inward prayer is positively associated with both ruminative and confronting IDs, which in turn are negatively related to well-being.

In our opinion, inward prayer, which centers on honest self-evaluation and the explicit expression of one’s flaws, can involve ruminative and confronting IDs. In ruminative IDs, people invoke difficult topics in one’s own mind and delve into them. As a result, such IDs lead to frustration or internal breakdown. Confronting IDs consist in playing internal conflicts in the form of a dialogue between two clearly separated parts of the self (Oleś 2009). Taking this into account, one can predict that these both types of ID will tend to decrease well-being.

#### H2

Upward prayer is positively associated with identity IDs which in turn is positively related to well-being.

Religion offers a distinctive sacred worldview, which can be strengthened in prayer. This can increase the sense of being part of a religious community, and, consequently, a social identity can be enhanced (cf. Park and Slattery 2013). In line with this, we assumed that upward prayer, which seeks to connect the person praying with the divine, can involve identity IDs aiming to answer questions such as “Who am I in relation to God?”, “What is the ultimate meaning of my life?”, and “How does God manifest in my neighbor?” These IDs result in enhancing a religious worldview and clarifying social (religious) identity, which in turn can have a positive effect on well-being.

#### H3

Outward prayer is positively associated with supportive IDs which in turn is positively related to well-being.

One of the most obvious benefits to those involved in organized religion is the social support that comes with that involvement (Park and Slattery 2013). Outward prayer that is a prayer for the intentions of other people can be seen as a type of involvement in a social network or a type of interaction with network members. At the same time, perceived social support has long been demonstrated to promote mental health. Perez et al. (2011) observed that cancer patients who pray for others experienced less depressive symptoms because they perceived greater social support and a deep interconnectedness with others. According to Prati and Pietrantoni (2010), believing that one can count on someone’s help is a better predictor of well-being than actual social support. Taking this into account, it seems that the link between outward prayer and well-being can be positively mediated through IDs reflecting social support (supportive dialogues).

## Method

### Respondents and Procedure

The study was conducted in Poland with a sample of 193 adults, 143 women and 50 men, aged between 16 and 60 years. The mean age was 24.62 years (SD = 7.66). Most participants were single, had at least secondary school education, and identified themselves as Roman Catholic. Table 1 presents additional demographic characteristics of the participants. The data were collected through a web survey, and the procedure was approved by the Research Ethics Committee at the Institute of Psychology at the authors’ university. The informed consent of the participants was implied through survey completion. Three measures were used in the following order: the Prayer Thoughts Scale, the Internal Dialogical Activity Scale, and the Psychological Well-Being Scale.

**Table 1:** Demographic characteristics of 193 respondents

Characteristic	*N*	%
*Sex*		
Female	143	74.1
Male	50	25.9
*Confession*		
Catholic	188	97.4
Orthodox	2	1.0
Protestant	3	1.6
*Education*		
Elementary	6	3.1
Vocational	6	3.1
Secondary	146	75.7
Higher	35	18.1
*Marital status*		
Single	178	92.2
Married	14	7.3
Widow	1	0.5
*Place of residence*		
Village	70	36.3
City or town < 200.000 people	58	30.1
City > 200.000 people	65	33.6
Total	193	100.0

### Measures

#### The Prayer Thoughts Scale (PTS)

This 29-item questionnaire by Ladd and Spilka (2002, 2006) was applied for the measurement of inward, outward, and upward directions of cognitive activities during prayer. The Inward scale, which involves self-evaluation, consists of the Examination (five items; e.g., *judging myself*) and Tears (three items; e.g., *misery*) subscales. The Upward scale, which involves adoration of the divine, consists of the Rest (four items; e.g., *quietude*) and Sacramental (three items; e.g., *connecting with traditions*) subscales. The Outward scale, which involves the physical world and its inhabitants, consists of Radical (four items; e.g., *boldness*), Suffering (three items; e.g., *agonizing with others*), Intercession (three items; e.g., *searching on behalf of someone else*), and Petition (four items; e.g., *requesting material things*) subscales (Ladd and Spilka 2002, 2006). The survey instruction directs participants to rate the degree to which each of the PTS items relates to their own thinking while engaged in prayer. Response options were from 1 (*strongly unrelated*) to 6 (*strongly related*). The scales and subscales are scored as a mean of their individual item scores. Convergent and divergent validity of the individual subscales of the PTS were confirmed (Ladd and Spilka 2006). The internal consistency for the Inward, Upward, and Outward scales analyzed in the current study is presented in Table 2.

**Table 2:** Prayer, internal dialogues, and well-being: correlations, reliability, means, and standard deviations

		1	2	3	4	5	6	7	8	9	10	11
1	Inward	–										
2	Upward	.59***	–									
3	Outward	.74***	.72***	–								
4	Pure	.05	.08	.06	–							
5	Identity	.13	.16*	.08	.59***	–						
6	Supportive	.09	.17*	.15*	.60***	.74***	–					
7	Ruminative	.26***	.05	.23**	.43***	.46***	.55***	–				
8	Confronting	.19**	.03	.08	.53***	.62***	.63***	.67***	–			
9	Simulation	.18*	.07	.17*	.53***	.34***	.46***	.37***	.34***	–		
10	Perspective	.15*	.08	.11	.58***	.63***	.67***	.49***	.67***	.39***	–	
11	Well-being	.05	.19**	.05	.02	.16*	.08	− .29***	− .16*	− .09	.09	–
	*M*	3.72	3.76	3.55	3.04	3.18	3.04	2.70	2.79	3.66	2.82	3.70
	SD	0.89	0.99	0.74	0.72	0.85	0.71	0.74	0.89	0.79	0.79	0.49
	*α*	.81	.84	.82	.62	.83	.72	.82	.78	.83	.74	.80

* *p* < .05; ** *p* < .01; *** *p* < .001

#### The Internal Dialogical Activity Scale (IDAS)

This questionnaire by Oleś (2009) is based on the assumption that the intensity of engaging in IDs is a trait-like personality disposition that can be measured according to the individual differences approach. An internal dialogical activity is defined in terms of engagement in dialogues with imagined figures, continuation, or simulation of social dialogical relationships in one’s own mind, and juxtaposing of the viewpoints relevant for personal and/or social identity (e.g., “I-as a believer” vs. “I-as a doubter”; Puchalska-Wasyl et al. 2008).

The IDAS consists of 47 items (including one buffer item, no. 1) that are designed in a Likert-type format with five alternative answers, from 1 (*I strongly disagree*) to 5 (*I strongly agree*). The IDAS measures the intensity with which the respondent conducts seven types of ID (Oleś 2009): (1) Pure Dialogical Activity—IDs conducted spontaneously; thinking and resolving problems in a dialogical form (e.g., *I converse with myself*); (2) Identity Dialogue—IDs aimed at better self-knowledge and at answering identity questions: *Who am I?, What is important to me?,* and *What is the meaning of my life* (e.g., *Sometimes I debate with myself about who I really am*); (3) Supportive Dialogue—IDs that confirm the possessed beliefs and provide support and a sense of being understood by the imagined interlocutor; substituting real conversations with imaginary ones; giving instructions to oneself (e.g., *In some stressful situations, I attempt to calm myself with my thoughts*); (4) Ruminative Dialogue—IDs on unpleasant issues; invoking difficult topics in one’s own mind and delving into them in the form of a dialogue; feelings of weariness, frustration, and internal breakdown related to internal dialogical activity (e.g., *After failures, I blame myself in my thoughts and discuss how the failures could have been avoided*); (5) Confronting Dialogue—IDs between two clearly separated parts of oneself, playing internal conflicts in the form of a dialogue (e.g., *Sometimes I think that my “good” side argues with my “bad” side*); (6) Social Simulation Dialogue—IDs that are a continuation or imagination of dialogical social relations: quarrels, discussions, or exchange of ideas (e.g., *Sometimes I continue a conversation with other people in my mind*); (7) Perspective-Taking Dialogue—IDs in which one is adopting a different view from one’s own point of view, i.e., a viewpoint of another person or a different part of one’s own self; objectivizing problems by looking at them from a different perspective (e.g., *Often in my thoughts I use the perspective of someone else*).

The subscales are scored as a mean of their individual item scores. We did not analyze the IDAS total score, which is the mean of all the subscale scores. In previous studies the internal consistency and stability of the IDAS measured at a two-month interval were high (*α* = .93; *r*_*tt*_ = .88; Oleś 2009). Cronbach’s alpha for the subscales ranged from .64 to .82. The internal consistency for the subscales established in the current study is presented in Table 2. The theoretical validity and construct validity of the IDAS were also confirmed (Oleś 2009).

#### The Psychological Well-Being Scale (PWBS)

This scale by Ryff (1989) contains 18 items rated on a six-point Likert scale, from 1 (*strongly disagree*) to 6 (*strongly agree*). The items reflect the six aspects of psychological well-being: Autonomy, Environmental Mastery, Personal Growth, Positive Relations with Others, Purpose in Life, and Self-Acceptance. Each aspect (subscale) is represented by three items. Example items are: *I judge myself by what I think is important, not by the values of what others think is important* (Autonomy); *I am quite good at managing the many responsibilities of my daily life* (Environmental Mastery); *For me, life has been a continuous process of learning, changing, and growth* (Personal Growth). We used a Polish adaptation of the PWBS (Cieciuch 2011; Karaś et al. 2013). In the current study, we used only the total score that measures the overall well-being, because five out of six subscales had unsatisfactory internal consistency: Autonomy (*α* = .55), Environmental Mastery (*α* = .62), Personal Growth (*α* = .46), Positive Relations with Others (*α* = .52), Purpose in Life (*α* = .33), and Self-Acceptance (*α* = .71). The internal consistency for the PWBS total score obtained in this study is presented in Table 2.

### Statistical Analysis

**Fig. 1:**
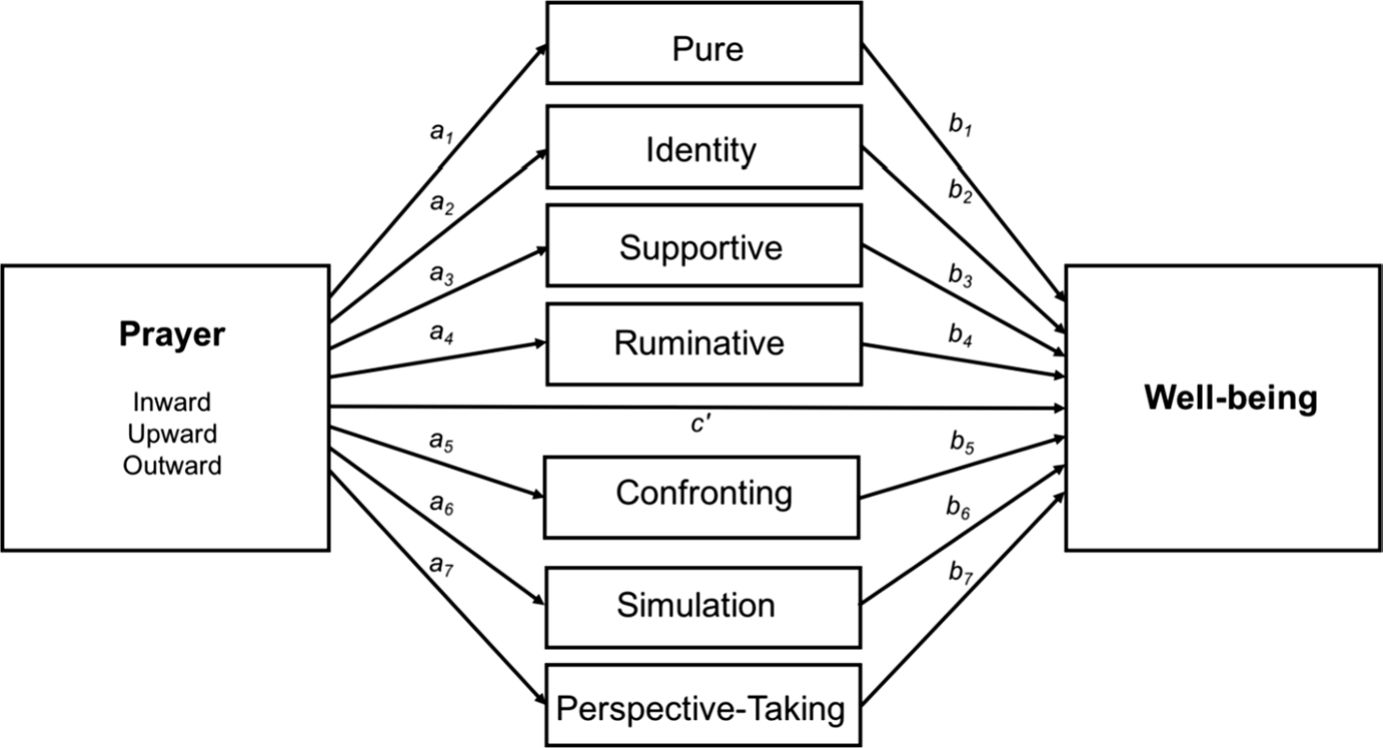
Conceptual model of how internal dialogues can mediate the effect of prayer on well-being. *c*′—direct effect of the predictor on the outcome while controlling for the mediator; *a*_*1*_*, a*_*2*_*, a*_*3*_*, a*_*4*_, *a*_*5*_*, a*_*6*_*, a*_*7*_—effects of the predictor on the mediator; *b*_*1*_*, b*_*2*_*, b*_*3*_*, b*_*4*_, *b*_*5*_*, b*_*6*_*, b*_*7*_—effects of the mediator on the outcome

We assessed the correlations among the key constructs of prayer, IDs, and well-being. To this end, zero-order correlations were performed between the PTS, IDAS, and PWBS. In a regression model, the prayer subscales (Inward, Upward, and Outward) were examined for their relationship to well-being. Seven types of IDs were tested as mediators in these relationships. Figure 1 shows the conceptual mediation model.

We performed all mediation analyses using PROCESS, model 4 (Hayes 2018). PROCESS is a path-analytic macro based on regression and estimates indirect effects and bias-corrected confidence intervals. We tested the significance of indirect effects using the bootstrapping procedure. Unstandardized indirect effects were computed for each of the 5000 bootstrapped samples and the corresponding 90% confidence intervals were computed. Other analyses were performed using SPSS v.24.

## Results

Prior to the main analysis, the assumptions of normality were tested using the Kolmogorov–Smirnov test with Lilliefors correction. The scores in the PTS as well as in two subscales of the IDAS (Identity and Social Simulation Dialogues) were slightly negatively skewed (from − 0.23 to − 0.74), while the other five subscales of the IDAS (Pure Dialogical Activity, Supportive, Ruminative, Confronting, and Perspective-Taking Dialogues) were slightly positively skewed (from 0.07 to 0.45). All of the coefficients of skewness were in the range from − 1 to 1, so the skewness was not strong enough and could be ignored (George and Mallery 2010). The scores in the PWBS were normally distributed.

### Correlation Analysis

Next, we calculated descriptive statistics and Pearson bivariate correlations for all variables examined in the regression model (Table 2). We observed the following significant positive correlations between prayer and IDs: Inward correlated with Ruminative, Confronting, Social Simulation and Perspective-Taking Dialogues; Upward correlated with Identity and Supportive Dialogues; and Outward correlated with Supportive, Ruminative, and Social Simulation Dialogues. Upward prayer was the only type of prayer that correlated positively with well-being. There were also quite strong correlations between the PTS subscales.

### Mediation Analysis

We performed three mediation analyses. In each of them, one type of prayer (inward, upward, or outward) was tested as a predictor of well-being, whereas seven types of ID were tested as parallel mediators in these relationships. The multicollinearity problem was identified in our model: The lowest tolerance index was 0.33, and the highest variance inflation factor (VIF) was 3.01. In order to manage this problem, when one type of prayer was treated as a predictor, two other types of prayer were introduced as covariants in each analysis. Significant outcomes of mediation analyses are presented in Table 3, whereas insignificant outcomes are presented in the supplementary material. The analyses indicated that there was a significant negative indirect effect of inward prayer on well-being through ruminative (*ab* = − .05, 90% CI [− .096, − .016]) and confronting dialogues (*ab* = − .05, 90% CI [− .099, − .011]): Inward prayer was positively associated with both ruminative (*a* = .20, 90% CI [.059, .341]) and confronting dialogues (*a* = .29, 90% CI [.116, .467]), which in turn were negatively related to well-being (*b* = − .27, 90% CI [− .372, − .171], *b* = − .17, 90% CI [− .261, − .070], respectively). There was also a significant positive indirect effect of upward prayer on well-being through identity (*ab* = .03, 90% CI [.003, .056]) and ruminative dialogues (*ab* = .05, 90% CI [.013, .101]): Upward prayer was positively associated with identity dialogues (*a* = .17, 90% CI [.023, .315]) which in turn were positively related to well-being (*b* = .15, 90% CI [.054, .251]); at the same time, upward prayer was negatively associated with ruminative dialogues (*a* = − .19, 90% CI [− .312, − .068]) which in turn were negatively related to well-being (*b* = − .27, 90% CI [− .372, − .171]). Finally, there was a significant negative indirect effect of outward prayer on well-being through ruminative dialogues (*ab* = − .06, 90% CI [− .029, − .060]): Outward prayer was positively associated with ruminative dialogues (*a* = .23, 90% CI [.035, .428]) which in turn were negatively related to well-being (*b* = − .27, 90% CI [− .372, − .171]). While controlling for the mediators, all the direct effects of inward, upward, and outward prayer on well-being were insignificant.

**Table 3:** Significant outcomes of mediation analyses from inward, upward, outward prayer to well-being assessing indirect effects of internal dialogues

Model	*R*^*2*^	*c’*	*a*	*b*	*ab*	90% CI	
						Lower	Upper
In–Rum–Wb	.28***	.08	.20*	− .27***	− .05	− .096	− .016
In–Conf–Wb	.28***	.08	.29**	− .17**	− .05	− .099	− .011
Up–Ident–Wb	.28***	.04	.17^^^	.15*	.03	.003	.056
Up–Rum–Wb	.28***	.04	− .19*	− .27***	.05	.013	.101
Out–Rum–Wb	.28***	− .03	.23^^^	− .27***	− .06	− .029	− .060

In, Inward; Up, Upward; Out, Outward; Rum, Ruminative Dialogues; Conf, Confronting Dialogues; Ident, Identity Dialogues; Wb, Well-being; *c*′, direct effect of predictor on outcome while controlling for the mediators; *a*, effect of the predictor on the mediator; *b*, effect of the mediator on the outcome; *ab*, indirect effect of predictor on outcome through the mediator; *R*^*2*^, amount of variance explained by the model; *CI*, confidence intervals

^^^ *p* < .10; * *p* < .05; ** *p* < .01; *** *p* < .001

## Discussion

The aim of this study was to determine the function of IDs in mediating the relationship between prayer and psychological well-being. While it is well established that prayer can support well-being, what factors determine whether prayer leads to an increase or a decrease in psychological well-being is less known. We attempted to answer this question by studying the relationships between inward, upward, and outward prayer and well-being, with IDs as mediators in these relationships. Specifically, we hypothesized that inward prayer can be negatively associated with well-being through ruminative and confronting IDs (H1); upward prayer can be positively related to well-being through identity IDs (H2); and outward prayer can be positively associated with well-being through supportive IDs (H3). Our expectations have been entirely confirmed regarding inward and upward prayer, and not confirmed with reference to outward prayer. Additionally, what was not posed in hypotheses, we found that the relationship between upward prayer and well-being was mediated by ruminative IDs which were negatively related to both these variables.

As only upward prayer turned out to have a positive link with well-being, we will first discuss this type of prayer. In accordance with our hypothesis, upward prayer is conducive to identity IDs and thus can support well-being. Upward prayer is concerned with the human–divine relationship, and explicitly seeks to connect the practitioner with the divine (Ladd et al. 2007). This orientation to God makes upward prayer the most “religious-rooted” form of prayer. Therefore, its link with identity IDs fits into the function of religion in shaping or strengthen social (religious) identity. Upward prayer, triggering identity questions such as “Who am I in the relation to God?” and “What is the ultimate meaning of my life?”, seems to contribute to shaping a distinctive sacred worldview as well as a bond with God and other believers (religious identity). Consequently, this might enhance well-being, since as Park and Slattery (2013) claimed: One of the pathways through which religiousness influences mental health outcomes is through the strong social identity that religion can offer.

As our results suggest, upward prayer can also reduce the chances of engaging in ruminative IDs and thus enhance well-being. Since ruminative IDs are related to ineffective problem-shooting and being tormented by them rather than seeking a constructive solution (Oleś 2009), they may coexist with reduced well-being, which is confirmed by the significant negative correlation between these two variables obtained in our study (Table 2). In contrast, upward prayer was the only type of prayer that correlated positively with well-being. Upward prayer which involves adoration of the divine (Ladd et al. 2007) shares much in common with the long-term, decision-based, committed approach to love. The emphasis here is not on ephemeral emotions or physicality. Instead, this reflects a rational, intentional choice (Ladd 2017). Presumably, the path of upward prayer–ruminative IDs–well-being works by replacing negative self-focused attention with concentrating on the divine, on gratitude toward God (cf. Perez et al. 2011). Lambert et al. (2010) suggest that one of the ways in which prayer works is by shifting the emphasis from oneself to a partner in the relationship. It is also consistent with Spilka and Ladd’s (2014) statement that prayer enables people to isolate themselves from their problems. Temporarily removing the problem from one’s mental field of view may allow one’s mental strength to regenerate, as well as enhance one’s sense of control over the situation. Consequently, this may induce an increase in well-being, as suggested by our study.

Taking into account inward prayer, the situation is completely different. In this type of prayer, emphasis is on honest self-evaluation and the explicit expression of one’s flaws. One form of inward prayer is an examination of conscience. Inward prayer often includes a component of tears, or an emotional reaction, especially when the examination centers on shortcomings (Ladd et al. 2007). Such type of prayer is very personal and demonstrates how a person reflects on his/her individual spiritual condition; it can be understood as dealing with the “internal concerns” of the self (Ladd 2017). In light of our results, when a person analyzes his/her own behavior in the presence of God during inward prayer, he/she seems to do it in two basic ways: He/she involves confronting and/or ruminative IDs. In the former case, while experiencing a dilemma or internal conflict, the person plays it out in the form of a dialogue between two clearly separated parts of oneself (e.g., “I-as blaming myself” vs. “I-as justifying myself”). In the latter case, the dialogue does not have to be conducted between two viewpoints treated as one’s own: For example, one part can be a personal point of view and the other can be God’s perspective. Regardless of their type, IDs associated with inward prayer concern difficult personal issues that are analyzed many times and cannot be solved constructively, which leads to frustration and internal breakdown. In our study, inward prayer was negatively related to well-being through ruminative and confronting IDs. This is consistent with Sedek and Kofta’s (1990) thinking that, when the problem seems to be impossible or difficult to solve, the adverse situation reduces the ability of a person to think flexibly. Ruminative and confronting IDs can be the manifestation of such stiffness. The cognitive rigidity makes it impossible to deal with the problem effectively. Delving into the problem inefficiently causes a state of frustration and cognitive exhaustion (Sedek et al. 1993), which can result in decreased well-being, as suggested by our study.

We hypothesized that outward prayer would be positively associated with supportive IDs which in turn would be positively related to well-being. Contrary to our expectations, the hypothesis was not confirmed. It turned out instead that outward prayer, similarly to inward prayer, is accompanied by ruminative IDs which have a negative link with well-being. Outward prayer is characterized by the emphasis on connecting with physical relational life. The most intense component of outward prayer is when the cognitive content centers on the intentional desire to join in the suffering of the other. A hallmark of this type of prayer is the request to be so “present” as to feel another’s pain (Ladd et al. 2007). This suggests a strong sense of the “other” in outward prayer (Ladd 2017). The fact that such prayer is rooted in the pain of the other person important to oneself probably intensifies the prayer and makes it persistent and even stubborn. However, the longer the prayer is seen as not answered by God, the more it gains ruminative characteristics. Stiffness of prayer, mentioned while discussing inward prayer, associated with the conviction that God does not care about us, finally leads to frustration and internal breakdown. Presumably, this is why outward prayer connected with ruminative IDs could lower well-being.

Although our hypotheses were only partially confirmed, this study generally shows that some types of ID actually mediate the relationship between prayer and well-being. First, this suggests that prayer can be treated as a dialogical phenomenon. It is in line with the fact that many authors emphasized communicative aspects of prayer (Baesler 2003; Beach et al. 2008; Lambert et al. 2010). It is also consistent with our thinking that on an observational level personal prayer can take a form of intrapersonal communication, although it should not be reduced to any type of ID. Second, the study also makes us aware of the important role of IDs which epitomize the idea of intrapersonal communication. Additionally, this role is very complex, which means that IDs perform different functions depending on their type and the context in which they appear. For example, ruminative IDs always trigger difficult questions and lead to feelings of weariness, frustration, and internal breakdown. However, ruminative IDs associated with inward prayer are mainly used for self-blaming, while the ruminative IDs related to outward prayer are an expression of the persistent striving for the good of the other person. Further research is needed to explore the impact of IDs on well-being in the context of different phenomena.

With regard to the shortcomings of the study, it should be stressed that the cross-sectional, non-experimental design limits our ability to draw causal conclusions about the findings. For example, we cannot be sure whether well-being is the result or rather the cause of upward prayer, which reduces ruminative IDs. Moreover, in the present study we have treated type of prayer as a predictor of well-being and ID as a mediator of this relationship. However, in other study we (Puchalska-Wasyl and Zarzycka 2019) tested a model in which ID was a predictor and well-being was an outcome, with upward, inward, and outward prayer being parallel mediators. We found that upward prayer worked as a mediator of the relationship between ID and well-being: Different types of ID, which (according to the model) turned into upward prayer, increased well-being. The effects obtained ranged from .05 to .11. In the present study, the effects are lower: from .03 to − .06. Taking stronger mediation paths into account, the model previously tested can be seen as more probable, but in our opinion it is conceivable that both models can reflect reality to some degree. In order to solve the problem of influence directions, an experimental research is needed. When it comes to further limitations of our study, post hoc power analyses showed that in three models tested in this paper the statistical power of Pure Dialogical Activity (.06), Supportive Dialogues (.22 to .26), and Social Simulation Dialogues (.19 to .21), along with the respective indirect effect sizes (.01 or less for the three variables in the three models tested), were small. Therefore, we cannot definitely conclude that these types of ID are not mediators of the relationship between inward, upward, and outward prayer and well-being; we cannot also rely on the assessment of indirect effect sizes of these three types of ID. Studies conducted with a bigger sample should be performed to examine this in much more depth. Another limitation is that our study was based on individual self-reports, and thus, the response bias could not be controlled. However, this problem was probably attenuated by the fact that participants completed the questionnaires anonymously. Furthermore, the sample was dominated by women and by Polish Roman Catholics; therefore, the results need to be replicated with samples where the current shortcomings are minimized.

## Conclusions

Taken together, this study aimed to examine whether and how IDs can be mediators of the relationship between upward, inward, and outward prayer and well-being. Depending on the type of ID that people conduct during their prayers, we could observe an increase or decrease in well-being. Inward prayer accompanied by ruminative and confronting IDs, as well as outward prayer accompanied by ruminative IDs, were negatively related to well-being. By contrast, upward prayer which was positively related to identity IDs and negatively to ruminative IDs was conducive to well-being. The results should be replicated in studies where the limitations of the current study will be minimized.

## Electronic supplementary material

Below is the link to the electronic supplementary material.

Supplementary material 1 (DOCX 15 kb)
