# Supplementary material for: Internal Dialogue as a Mediator of the Relationship Between Prayer and Well-Being
Source: J Relig Health. 2019 Nov 9;59(4):2045–63. doi: 10.1007/s10943-019-00943-2 (PMC7359138; doi:10.1007/s10943-019-00943-2)
Supplement: Supplementary file 1 — Supplementary material 1 (DOCX 15 kb) [file 10943_2019_943_MOESM1_ESM.docx]

Supplemental File

*Insignificant Outcomes of Mediation Analyses from Inward, Upward, and Outward Prayer to Well-being Assessing Indirect Effects of Internal Dialogues*

| Model | *R^2^* | *c’* | *a* | *b* | *ab* | 90% CI | |
| --- | --- | --- | --- | --- | --- | --- | --- |
|  |  |  |  |  |  | Lower | Upper |
| In–Pure–Wb | .28*** | .08 | <.01 | .01 | <.01 | -.012 | .011 |
| In–Ident–Wb | .28*** | .08 | .12 | .15* | .02 | -.009 | .050 |
| In–Sup–Wb | .28*** | .08 | -.05 | .13 | -.01 | -.028 | .015 |
| In–Simul–Wb | .28*** | .08 | .12 | -.07 | -.01 | -.033 | .004 |
| In–Persp–Wb | .28*** | .08 | .13 | .13* | .02 | -.003 | .047 |
| Up–Pure–Wb | .28*** | .04 | .06 | .01 | <.01 | -.011 | .011 |
| Up–Sup–Wb | .28*** | .04 | .09 | .13 | .01 | -.003 | .034 |
| Up–Conf–Wb | .28*** | .04 | -.08 | -.17** | .01 | -.009 | .044 |
| Up–Simul–Wb | .28*** | .04 | -.10 | -.07 | .01 | -.004 | .026 |
| Up–Persp–Wb | .28*** | .04 | -.02 | .13* | <-.01 | -.019 | .015 |
| Out–Pure–Wb | .28*** | -.03 | <-.01 | .01 | <.01 | -.013 | .017 |
| Out–Ident–Wb | .28*** | -.03 | -.17 | .15* | -.03 | -.077 | .012 |
| Out–Sup–Wb | .28*** | -.03 | .10 | .13 | .01 | -.019 | .047 |
| Out–Conf–Wb | .28*** | -.03 | -.08 | -.17** | .01 | -.029 | .060 |
| Out–Simul–Wb | .28*** | -.03 | .17 | -.07 | -.01 | -.042 | .009 |
| Out–Persp–Wb | .28*** | -.03 | .02 | .13* | <.01 | -.026 | .034 |

*Note*. In = Inward; Up = Upward; Out = Outward; Pure = Pure Dialogical Activity; Ident = Identity Dialogues; Sup = Supportive Dialogues; Simul = Social Simulation Dialogues; Persp = Perspective-Taking Dialogues; Conf = Confronting Dialogues; Wb = Well-being; *c’* = direct effect of the predictor on the outcome while controlling for the mediator; *a* = effec t of the predictor on the mediator; *b* = effect of the mediator on the outcome; *ab* = indirect effect of predictor on outcome through the mediator; *R^2^* = amount of variance explained by the model; *CI* = confidence intervals

^^^ *p* < .10; ^*^*p* < .05; ^**^*p* < .01; ^***^*p* < .001
